# MYH9 Aggregation Induced by Direct Interaction With PRRSV GP5 Ectodomain Facilitates Viral Internalization by Permissive Cells

**DOI:** 10.3389/fmicb.2019.02313

**Published:** 2019-10-09

**Authors:** Biyun Xue, Gaopeng Hou, Guixi Zhang, Jingjing Huang, Liangliang Li, Yuchen Nan, Yang Mu, Lizhen Wang, Lu Zhang, Ximeng Han, Xiaolei Ren, Qin Zhao, Chunyan Wu, Jingfei Wang, En-Min Zhou

**Affiliations:** ^1^College of Veterinary Medicine, Northwest A&F University, Xianyang, China; ^2^State Key Laboratory of Veterinary Biotechnology, Harbin Veterinary Research Institute, Chinese Academy of Agricultural Sciences, Harbin, China

**Keywords:** MYH9, PRRSV, GP5, protein–protein interaction, virus internalization

## Abstract

Prevention and control of infection by porcine reproductive and respiratory syndrome virus (PRRSV) remains a challenge, due to our limited understanding of the PRRSV invasion mechanism. Our previous study has shown that PRRSV glycoprotein GP5 interacts with MYH9 C-terminal domain protein (PRA). Here we defined that the first ectodomain of GP5 (GP5-ecto-1) directly interacted with PRA and this interaction triggered PRA and endogenous MYH9 to form filament assembly. More importantly, MYH9 filament assembly was also formed in GP5-ecto-1-transfected MARC-145 cells. Notably, PRRSV infection of MARC-145 cells and porcine alveolar macrophages also induced endogenous MYH9 aggregation and polymerization that were required for subsequent PRRSV internalization. Moreover, overexpression of S100A4, a MYH9-specific disassembly inducer, in MARC-145 cells significantly resulted in diminished MYH9 aggregation and marked inhibition of subsequent virion internalization and infection by both *PRRSV-1* and *PRRSV-2* isolates. The collective results of this work reveal a novel molecular mechanism employed by MYH9 that helps PRRSV gain entry into permissive cells.

## Introduction

Porcine reproductive and respiratory syndrome virus (PRRSV) is a positive-stranded, enveloped RNA virus belonging to the formerly designated genus *Arterivirus* (Nan et al., 2017). Based on a recent proposal, all PRRSV isolates have been assigned to two species within the genus *Porartevirus*, *PRRSV-1* and *PRRSV-2*, as part of a recently revised taxonomic scheme (Adams et al., 2016; Kuhn et al., 2016). Viral isolates from *PRRSV-1* and *2* species share approximately 60% nucleotide sequence similarity and exhibit serotype differences (van Woensel et al., 1998; Forsberg, 2005). The genome size of PRRSV is approximately 15 kb and contains at least 10 open reading frames (ORFs) (Lunney et al., 2016). ORF1a and ORF1b account for two-thirds of the entire PRRSV genome and encode the replicase required for viral replication, while ORFs 2–7 encode most structural proteins involved in virion assembly (Lunney et al., 2016).

Porcine reproductive and respiratory syndrome virus GP5 consists of approximately 200 amino acids. Based on the predicted model derived from the GP5 amino acid sequence, the GP5 protein is structurally organized into three or four predicted transmembrane domains and possesses an N-terminal cleavable signal peptide that directs protein synthesis to the rough ER (Veit et al., 2014). The ectodomain of GP5 consists of 35 amino acids that include several putative N-glycosylation sites comprising a neutralizing antibody epitope that is broadly shared by PRRSV isolates (Ansari et al., 2006). GP5 has been demonstrated to be indispensable for virus particle assembly and replication within susceptible cells (Wissink et al., 2005; Ansari et al., 2006; Firth et al., 2011). Meanwhile, the complex formed by GP2a and GP4 has been shown to interact with CD163 (Brock et al., 2012; Tian et al., 2012), an essential host receptor for PRRSV infection both *in vitro* and *in vivo* (Whitworth et al., 2016; Burkard et al., 2017, 2018; Yang et al., 2018). However, the virus infection process may involve additional cell receptors, as other studies have suggested that the GP5/M complex binds sialoadhesin (CD169) (Van Breedam et al., 2010; Thaa et al., 2017), another putative receptor involved in PRRSV entry (Delputte et al., 2007). Notably, transgenic pigs lacking sialoadhesin are still susceptible to viral infection, implying that a GP5/M-sialoadhesin interaction is not necessary for PRRSV entry into permissive cells (Prather et al., 2013). Due to these conflicting results, the cellular partners that interact with GP5 and the role that GP5 plays during PRRSV infection remain unclear.

Our previous studies had demonstrated that an anti-idiotypic monoclonal antibody (Mab2-5G2), which is specific for an idiotypic antibody to PRRSV GP5, recognizes the C-terminal domain of non-muscle myosin heavy chain 9 (MYH9) found in PRRSV-permissive cells. Thus, this fact, together with the known indispensable role of MYH9 in host cell internalization of PRRSV, both suggest that MYH9 likely serves as a novel host factor involved in PRRSV infection (Zhou et al., 2008; Gao et al., 2016). Besides PRRSV, MYH9 has been identified as a cellular receptor for herpes simplex virus-1 (HSV-1) (Arii et al., 2010), severe fever with thrombocytopenia syndrome virus (SFTSV) (Sun et al., 2014), and Epstein-Barr virus (EBV) (Xiong et al., 2015). Notably, we have recently demonstrated that PRRSV GP5 interacts with the C-terminal domain of MYH9 (hereafter referred to as PRA) and that interruption of the GP5-MYH9 interaction via addition of recombinantly expressed PRA blocked both PRRSV infection of permissive cells and later virus replication within those cells *in vitro* (Li et al., 2018).

MYH9 possesses both an N-terminal motor domain, which regulates actin network assembly, and a C-terminal cargo-binding tail domain (Avisar et al., 2008). It is believed that MYH9 is recruited as a bridge protein that links actin to internalized viral particles present within endosomal vesicles, ultimately promoting transport of vesicular cargo along with actin filaments into the cytoplasm. This process requires myosin activity and actin polymerization and facilitates entry of larger virus particles (Cureton et al., 2009, 2010; Piccinotti et al., 2013) by bending internal and external membranes to enable internalization. During virus infection of susceptible cells, such as with PRRSV (enveloped virus) and bovine parvovirus (non-enveloped virus), complete virion internalization involves interactions between viral proteins and cellular receptors, as well as participation of clathrin-mediated endocytosis aided by MYH9, an essential factor (Dudleenamjil et al., 2010; Arii et al., 2015; Xiong et al., 2015; Gao et al., 2016). Previously it had been reported that myosin participates in virus assembly and release by transporting viral structural proteins (Kumakura et al., 2015) or by regulating endosomal recycling (Varthakavi et al., 2006). More recently, MYH9 has also been demonstrated to act as a negative regulator during early viral infection, whereby it recognizes sialic acids on sialylated RNA viruses then suppresses pro-inflammatory signaling via the DAP12-Syk pathway (Liu et al., 2019). However, the detailed mechanism of myosin involvement in PRRSV internalization of permissive cells remains elusive.

In this study, we used truncation analysis to first narrow down the GP5 region involved in the direct interaction between GP5 and PRA within the first ectodomain of GP5 (designated GP5-ecto-1). We then demonstrated that the GP5-ecto-1 directly interacted with the PRA region within the C-terminal domain of MYH9. Moreover, GP5-ecto-1 induced filamentous aggregates of recombinant PRA proteins *in vitro* and of endogenous MYH9 *in vivo*, which could be disassembled by the addition of regulatory protein S100A4. Upon further analysis, PRRSV GP5 was found to preferentially interact with the dimerized form of PRA, but not with S100A4-dissociated monomeric PRA. Notably, PRRSV infection induced MYH9 to form aggregates that facilitated viral internalization by both MARC-145 cells and PAMs. Conversely, endogenous S100A4 did not participate in MYH9-mediated endocytosis as reflected by the fact that mRNA and protein expression levels remained unchanged and the protein failed to co-localize with MYH9 aggregates during PRRSV internalization. Interestingly, introduction of S100A4 protein expression within MARC-145 cells significantly diminished MYH9 aggregation and ultimately inhibited PRRSV internalization and sequential infection regardless of PRRSV genotype. Taken together, our research revealed a novel molecular mechanism that is involved in both the GP5-MYH9 interaction and PRRSV invasion of permissive cells.

## Materials and Methods

### Cells and Viruses

Cell lines MARC-145 (simian kidney epithelial cells derived from MA-104) and COS7 (African green monkey kidney fibroblast-like cells) were purchased from the China Center for Type Culture Collection (CCTCC, Wuhan, China). Generation of MARC-145-GP5^*Flag*^ cells by lentiviral transduction was performed as previously described (Gao et al., 2016). Cells were cultured in Dulbecco’s Modified Eagle Medium (DMEM, Gibco, Carlsbad, CA, United States) supplemented with 10% fetal bovine serum (FBS, PAN-Biotech, Aidenbach, Germany), 100 U/mL of penicillin, and 100 mg/mL of streptomycin and incubated at 37°C with 5% CO_2_. Porcine alveolar macrophages (PAMs) were collected from specific-pathogen-free (SPF) pigs and maintained in RPMI 1640 medium (Gibco) supplemented with 10% FBS. To maintain continuous GP5 expression in MARC-145-GP5^*Flag*^ cells, complete DMEM medium containing 8 μg/mL puromycin (Thermo Fisher Scientific, Waltham, MA, United States) was used.

*PRRSV-2* strains used in this study included SD16 (GenBank ID: JX087437.1), JXA1 (GenBank: EF112445.1), VR2332 (GenBank: EF536003.1), and CH-1a (GenBank: AY032626). Two *PRRSV-1* isolates, GZ11-G1 (GenBank: KF001144.1) and P073-3 (only partially sequenced and confirmed to be a *PRRSV-1* isolate, full sequence is unavailable), were included as well. All PRRSV strains were propagated and titrated in MARC-145 cells as previous reported (Li et al., 2018).

### Co-immunoprecipitation Assay

MARC-145-GP5^*Flag*^ cells were cultured in 10-cm dishes. Cell monolayers were harvested by ultrasonication in cold PBS supplemented with protease inhibitor cocktail (Sigma-Aldrich) and clarified by centrifugation at 13,000 × *g* for 40 min at 4°C. Cell lysates of MARC-145-GP5^*Flag*^ cells were either treated with Peptide-N-Glycosidase F (PNGase F, New England Biolabs, Ipswich, MA, United States) or neuraminidase (New England Biolabs) according to the manufacturers’ instructions at 37°C for 1 h. Co-immunoprecipitation (Co-IP) assays were conducted using anti-Flag monoclonal antibody (Sigma-Aldrich, St. Louis, MO, United States) overnight and immune complexes were pulled down using Protein G Dynabeads (Thermo Fisher Scientific). After removing Co-IP supernatants and washing Dynabeads three times with PBS, Dynabeads were harvested by suspension in 5 × Loading Buffer (Beyotime, Jiangsu, China) then subjected to SDS-PAGE and Western blot analysis. Mouse IgG (mIgG, Beyotime, Jiangsu, China) was used as an antibody isotype control.

MARC-145 cells were cultured in 10-cm dishes and cell monolayers were transfected with plasmids encoding GP5-HA, GP5-N-HA, or GP5-C-HA. At 48 h after transfection, cells were lysed using NP-40 cell lysis buffer (Beyotime, Jiangsu, China) supplemented with a protease inhibitor cocktail (Sigma-Aldrich) and clarified as described above. Next, Co-IP was conducted using anti-HA monoclonal antibody (Sigma-Aldrich) following the same protocol described above.

To analyze the interaction between GP5 and different forms of PRA, COS7 cell monolayers were transfected with plasmid encoding GP5-HA. Next, transfected cells were lysed and clarified as described above then incubated with PRA (5 mM) and treated with or without S100A4 (10 mM, containing 3 mM CaCl^2+^) at 4°C for 1 h. Next, Co-IP was conducted using anti-HA monoclonal antibody (Sigma-Aldrich). Dynabeads incubated with antibody and pulled-down proteins were harvested by suspension in 5× non-reducing loading buffer (Aladdin, Shanghai, China) then subjected to SDS-PAGE and Western blot analysis.

### Prokaryotic Expression Plasmids Construction and Recombinant Proteins Expression

The cDNA sequence of full-length PRRSV-ORF5 (SD16) was obtained from the DNA sequence of infectious clone pBAC-PRRSV-SD16 as previous reported (Wang et al., 2013). The cDNA of GP5Δ and first ectodomain of GP5 (GP5-ecto-1, 32-66 aa) were cloned and inserted into *Bam*HI and *Xho*I sites of pET-28a that had been also used for GP5-C (126-200 aa) vector construction to allow bacterial expression as well. The cDNA encoding for S100 Ca^2+^-binding protein A4 (S100A4) was synthesized by GENEWIZ Co., Ltd. (Suzhou, Jiangsu, China) and directly ligated into *Bam*HI and *Xho*I sites of pGEX-6P-1 vector to express protein fused with the GST tag. Primers used for cloning of above plasmids are shown in Table 1.

**TABLE 1:** List of the primers used in this study.

**Primers name**	**Sequence (5′–3′)**	**Inserted Vector**	**Target gene**
GP5Δ-F1	AACGGATCCGCCAGCAACAACAACAGC	pET-28a	Cloning of GP5Δ-His
GP5Δ-R1	AGTCTCCACTGCCCAGTCA	pET-28a	Cloning of GP5Δ-His
GP5Δ-F2	AGGCTTGCGAAGAACTGCA	pET-28a	Cloning of GP5Δ-His
GP5Δ-R2	TACCTCGAGCTAGAGACGACCCCATTG	pET-28a	Cloning of GP5Δ-His
GP5Δ-F3	GACACAGTTGGTCTGGCCACTGTGTCCACCG	pET-28a	Cloning of GP5Δ-His
	CCGGATATTAT**GGCTCTTCC**AGGCTTGCGA^∗^		
GP5Δ-R3	ATAATATCCGGCGGTGGACACAGTGGCCAGAC	pET-28a	Cloning of GP5Δ-His
	CAACTGTGTC**GGAAGAGCC**AGTCTCCACT		
GP5-HA-F	GCCGAATTCATGTTGGGGAAGTGC	pCAGEN	Cloning of GP5-HA
GP5-HA-R	ATAGCGGCCGCCTAAGCGTAATCTGGAACAT	pCAGEN	Cloning of GP5-HA
	CGTATGGGTAGAGACGACCCCATTGTT		
GP5-N-HA-F	GCCGAATTCATGTTGGGGAAGTGC	pCAGEN	Cloning of GP5-N-HA
GP5-N-HA-R	ATAGCGGCCGCCTA*AGCGTAATCTGGAACATC*	pCAGEN	Cloning of GP5-N-HA
	*GTATGGGTA*ATAATACCGGCGGTGGACA		
GP5-C-HA-F	CTCGAATTCATGGTCATTAGGCTTGCG	pCAGEN	Cloning of GP5-C-HA
GP5-C-HA-R	ATAGCGGCCGCCTA*AGCGTAATCTGGAACATC*	pCAGEN	Cloning of GP5-C-HA
	*GTATGGGTA*GAGACGACCCCATTGTT		
GP5-ecto-1-His-F	GCCGGATCCAGCAACAACAACAGC	pET-28a	Cloning of GP5-ecto-1-His
GP5-ecto-1-His-R	TACCTCGAGCTAAGTCTCCACTGCCCAG	pET-28a	Cloning of GP5-ecto-1-His
GP5-C-His-F	AACGGATCCAGGCTTGCGAAGAAC	pET-28a	Cloning of GP5-C-His
GP5-C-His-R	TACCTCGAGCTAGAGACGACCCCATTG	pET-28a	Cloning of GP5-C-His
GP5-ecto-1-HA-F	GCCGAATTCATGAGCAACAACAACAGC	pCAGEN	Cloning of GP5-ecto-1-HA
GP5-ecto-1-HA-R	ATAGCGGCCGCCTA*AGCGTAATCTGGAACATC*	pCAGEN	Cloning of GP5-ecto-1-HA
	*GTATGGGTA*AGTCTCCACTGCCCAG		
S100A4-myc-F	ATAGAATTCGCCTACCCCCTGGAG	pCAGEN	Cloning of S100A4-myc
S100A4-myc-R	ATAGCGGCCGCTCA*CAGATCCTCTTCTGAGATG*	pCAGEN	Cloning of S100A4-myc
	*AGTTTTTGTTC*CTTCTTCCGGGGCTGCTTAT		
S100A4-F	TGAGCAACTTGGACAGCAACAGG	–	qPCR detection of S100A4 gene
S100A4-R	TTACACATCATGGCGATGCAGGAC	–	qPCR detection of S100A4 gene
aORF-7-F	ATGGCCGGTAAAAATCAGAGCC	–	qPCR detection of N gene of *PRRSV-1*
aORF-7-R	TTAATTCGCACCCTGACTGG	–	qPCR detection of N gene of *PRRSV-1*
bORF-7-F	ATGCCAAATAACAACGGCAAGCAGC	–	qPCR detection of N gene of *PRRSV-2*
bORF-7-R	TCATGCTGAGGGTGATGCTGTG	–	qPCR detection of N gene of *PRRSV-2*

*^∗^The bold letters are GSS sequence; Italic letters are HA tag and myc tag sequences.*

Expression of recombinant GP5Δ-His, GP5-ecto-1-His and GP5-C-His was obtained as previous described with the following modifications (Yuan et al., 2014). Briefly, pET-28a- GP5Δ-His, pET-28a-GP5-ecto-1-His and pET-28a-GP5-C-His plasmids were introduced into *E. coli* BL21 (DE3) cells and expression of recombinant proteins was induced with IPTG (0.3 mM) at 37°C for 6 h. After sonication, inclusion bodies were collected and dissolved in 8 M urea and 100 mM Tris, pH 8.0, followed by purification using His-Tag resin (Roche) and elution using 100 mM imidazole. Purified proteins were refolded by dialysis against buffer containing a gradient of urea concentrations (6, 4, 3, 2, 1, 0.5, and 0 M) until buffer was completely replaced by 100 mM Tris (pH 8.0). Recombinant proteins were further concentrated using 3 kDa ultrafiltration centrifugal tubes (EMD Millipore, Boston, MA, United States) and proteins were analyzed for purity before experimental use.

For recombinant proteins expressed in bacteria, expression and purification of the SUMO-tagged PRA region of the C-terminal domain of MYH9 (His-SUMO-PRA) was performed as previously described (Li et al., 2018). As necessary, the His-SUMO-tag could be removed by recombinant Tobacco Etch Virus protease (rTEV protease) expressed in-house as previously described (Li et al., 2018).

Expression and purification of recombinant S100A4 was conducted as previously described in *E. coli* BL21 (DE3) cells (Kiss et al., 2016). Briefly, expression of recombinant GST-S100A4 protein was induced with IPTG (0.3 mM) at 16°C for 20 h. Bacterial cells were collected by centrifugation and subjected to sonication. After removing cell debris, supernatant was purified using Glutathione Sepharose^®^ 4B Resin (GE Healthcare) for GST affinity purification and washed with Buffer A (50 mM Tris pH 8.0, 150 mM NaCl, 10% glycerol) for three times. Then S100A4 was separated from the GST tag after cleavage with PreScission protease (GE Healthcare) at 4°C overnight. The effluent containing S100A4 was further purified by RESOURCE Q anion exchange chromatography (GE Healthcare) at pH 5.5.

### Far-Western Blot Assay

To study the direct interaction between PRA and GP5Δ, GP5-ecto-1, far-Western blot analysis was conducted. Briefly, recombinantly expressed GP5Δ-His, GP5-ecto-1-His or PRA at the indicated concentration were resolved by SDS-PAGE and transferred to PVDF membranes as bait proteins followed by blocking of PVDF membranes with 5% skim milk in PBS. Next, membranes were probed with PRA or GP5-ecto-1-His or GP5Δ-His at a concentration of 5 μg/mL dissolved in PBS buffer for 2h at 37°C. The GP5Δ-His-PRA and GP5-ecto-1-PRA interaction were detected with Mab-5G2 or anti-His mAb, and visualized by HRP-labeled goat anti-mouse IgG (H + L) (Jackson Laboratories) followed by ECL substrate with a ChemiDoc^TM^ MP Imaging System (Bio-Rad Laboratories). Home-made recombinant swine hepatitis E virus ORF2 protein (p239) was used as negative protein control.

### Indirect Enzyme-Linked Immunosorbent Assays

Indirect enzyme-linked immunosorbent assays (IELISAs) were conducted to detect the interaction between recombinant PRA and GP5Δ. Briefly, 96-well polystyrene microtiter plates (Corning, NY, United States) was coated with CP5Δ-His or PRA at the indicated concentration overnight at 4°C, blocked with 5% skim milk in PBS-T buffer [PBS containing 0.5% Tween 20 (Sigma-Aldrich)] and incubated with PRA or CP5Δ-His for 1 h at 37°C. After washed by PBS-T buffer for three times, interaction was detected using anti-His or Mab2-5G2 antibodies, HRP-conjugated goat anti-mouse IgG antibody as secondary antibody and visualized using a 3,3′,5,5′-tetramethylbenzidine (TMB) kit (TianGen, Beijing, China). The values of absorbance at 450 nm were evaluated using a Victor^TM^ X5 Multilabel Plate Reader (PerkinElmer, Waltham, MA, United States). Protein p239 was used as negative protein control.

### Eukaryotic Expression Plasmids Construction and Transfection

Mammalian transient expression plasmids for transfection were constructed using cDNA encoding GP5, GP5-N (1-102 aa, containing signal peptide with both ectodomains), GP5-C (126-200 aa, containing cytoplasmic tail), GP5-ecto-1 (32-66 aa, containing the first ectodomain), or S100 Ca^2+^-binding protein A4 (S100A4) fused with HA-tag or myc-tag at carboxy-terminal ends. Insert DNA was cloned into *Eco*RI and *Not*I sites of the pCAGEN vector (Addgene plasmids number #11160). Synthesis of cDNAs coding for S100A4 was performed by GENEWIZ Co., Ltd. followed by direct ligation of cDNA inserts into pCAGEN. Primers used for cloning of abovementioned plasmids are shown in Table 1.

Transient transfections of MARC-145 or COS7 cells with eukaryotic expression plasmids were conducted using Lipofectamine^®^ 3000 Reagent (Thermo Fisher Scientific) according to the manufacturer’s instructions.

### Western Blot Analysis

Cells were lysed using ice-cold NP-40 lysis buffer (Beyotime) supplemented with protease inhibitor cocktail (Sigma-Aldrich) then mixed with 5 × Loading Buffer (Beyotime) for SDS-PAGE. Equal amounts of protein samples were loaded onto 12% SDS-PAGE gels and separated proteins were transferred onto PVDF membranes as described previously (Zhang et al., 2017). Membranes were blocked with 5% skim milk in PBS and probed with anti-MYH9 rabbit polyclonal antibody (Sigma-Aldrich), anti-HA monoclonal antibody (Sigma-Aldrich), anti-myc monoclonal antibody (Sigma-Aldrich), anti-His monoclonal antibody (Proteintech Group Inc., Rosemont, IL, United States), or two homemade antibodies that included an anti-PRRSV-nucleocapsid (N) monoclonal antibody (Mab-6D10, made in-house) and an anti-idiotypic monoclonal antibody recognizing MYH9 (Mab2-5G2) (Gao et al., 2016). Specific binding of antibodies to their targets was detected using affinity-purified goat anti-mouse or anti-rabbit IgG horseradish peroxidase (HRP) conjugates (Jackson Laboratories, West Grove, PA, United States) and visualized using ECL substrate (Beyotime). Chemiluminescence signal acquisition was conducted using a ChemiDoc MP imaging system (Bio-Rad Laboratories, Hercules, CA, United States) and analyzed using Image Lab software (Version 5.1, Bio-Rad Laboratories).

### His Pull-Down Assay

For the His pull-down assay, cell lysate supernatants containing GP5-HA, GP5-N-HA, or GP5-C-HA were incubated with His-SUMO-PRA-coated or His-SUMO-coated (as negative control) His-Tag protein-Trap resin (Roche, Basel, Switzerland) at 4°C overnight. Next, resins were washed with PBS three times and suspended in 5 × Loading Buffer (Beyotime) then subjected to SDS-PAGE and Western blot analyses.

To evaluate the interaction between GP5 and dimeric PRA via pull-down assay, His-SUMO-PRA- or S100A4-pretreated His-SUMO-PRA were first used to coat His-Tag protein-Trap resin (Roche) at 4°C overnight. Next, coated resins were washed with PBS three times before incubation with COS7 cell lysate containing GP5-HA. After incubation with lysate, resins were washed with PBS, suspended in 5 × Loading Buffer (Beyotime), then subjected to SDS-PAGE and Western blot analysis.

### Confocal Microscopy

MARC-145 cells were seeded onto coverslips and transfected with plasmids encoding GP5-N-HA, GP5-ecto-1-HA, GP5-C-HA, S100A4-myc, or empty vector (EV) for 48 h. To visualize PRRSV entry, non-transfected, as well as S100A4-myc or empty vector (EV)-transfected MARC-145 cells, were used for PRRSV inoculation. After exposure to PRRSV (MOI = 50) at 4°C for 2 h, MARC-145 cells were transferred to 37°C for 0, 5, 15, and 30 min.

After washing with PBS, cells were fixed in 4% paraformaldehyde (Sigma-Aldrich) for 10 min at room temperature and permeabilized using PBS containing 0.25% Triton X-100 (Sigma-Aldrich). Next, cells were probed with anti-HA monoclonal antibody, anti-MYH9 rabbit polyclonal antibody, anti-myc monoclonal antibody, anti-S100A4 goat polyclonal antibody (antibodies described above were all purchased from Sigma-Aldrich), or Mab-6D10 for 2 h at 37°C. After three washes, secondary antibodies (Thermo Fisher Scientific) that included Alexa Fluor^®^ 488-conjugated goat anti-mouse IgG (H + L), Cy3-conjugated goat anti-rabbit IgG (H + L), and Alexa Fluor^®^ 647-conjugated donkey anti-goat IgG (H + L), were incubated with cells for another 2 h at 37°C. Coverslips were mounted onto slides using ProLong^®^ Gold Antifade Reagent containing 4′,6-diamidino-2-phenylindole (DAPI) (Thermo Fisher Scientific) and observed under a confocal microscope (Leica Microsystems, Wetzlar, Germany). All images were captured and processed using Leica Application Suite X (Version 1.0., Leica Microsystems). Each Mander’s overlap coefficient, an indicator of co-localization, was determined using Image-Pro Plus software.

### Native-PAGE Analysis

Native-PAGE was performed as previously described with the following modifications (Walker, 1994). Briefly, cells were lysed in NP40 lysis buffer (Beyotime), and then subjected to native-PAGE (5% non-denaturing resolving gel with a 5% stacking gel) in running buffer (25 mM Tris, 192 mM glycine, and 1% deoxycholic acid sodium salt) for 2 h at 160 V. Separated proteins were transferred onto polyvinylidene fluoride (PVDF) membranes at 100 V for 4 h using a wet transfer protocol then membranes were blocked with PBS containing 5% skim milk for subsequent Western blot analysis.

### Electron Microscope

For negative staining, purified PRA (5 mM) alone or pre-incubated with GP5-ecto-1-His (10 mM) or GP5-C-His (10 mM), as well as recombinant S100A4 (10 mM) incubated with a complex of GP5-ecto-1-His/PRA or PRA, were incubated for 1 h at 4°C in buffer A (50 mM Tris, 150 mM NaCl, 10% glycerol) containing 3 mM CaCl_2_. After centrifugation at 15,000 rpm at 4°C for 10 min, sample supernatants were collected carefully. A total of 20 μL of each sample was applied to a carbon-coated grid that had been glow discharged (JFC-1600, JEOL, Japan) for 1 min in air. Next, grids were immediately negatively stained using 1% uranyl acetate then were examined using an H-7650 electron microscope (EM) (Hitachi, Tokyo, Japan) operating at 80 kV.

### RNA Isolation and Quantitative Real-Time PCR (qPCR)

Total RNA was extracted from cells using TRizol reagent (Invitrogen, Grand Island, NY, United States) according to the manufacturer’s instructions. Reverse transcription and qPCR were conducted using PrimeScript RT reagent Kit (TaKaRa, Dalian, China) and FastStart Universal SYBR Green Master (Roche) using a QuantStudio3 QPCR system (Applied Biosystems, Carlsbad, NY, United States). Transcripts of GAPDH were also amplified to normalize total RNA input. Relative quantification of target genes was performed using the 2^–Δ^ ^Δ^ ^*Ct*^ method. Primers used for amplifying the S100A4 mRNA sequence and N gene sequences of *PRRSV-1* (aORF-7) and *PRRSV-2* (bORF-7) isolates are shown in Table 1.

### Statistical Analysis

Unless otherwise indicated, all data are presented as the mean ± SD with error bars and representative data of at least three independent experiments are shown. Statistical analysis was performed using GraphPad Prism version 5.0 (GraphPad Software, San Diego, CA, United States). Differences in indicators between treatment groups and controls were assessed using the Student’s *t*-test. *P* < 0.05 was considered statistically significant.

## Results

### PRRSV GP5-MYH9 Interaction Does Not Require GP5 Glycosylation

**FIGURE 1:**
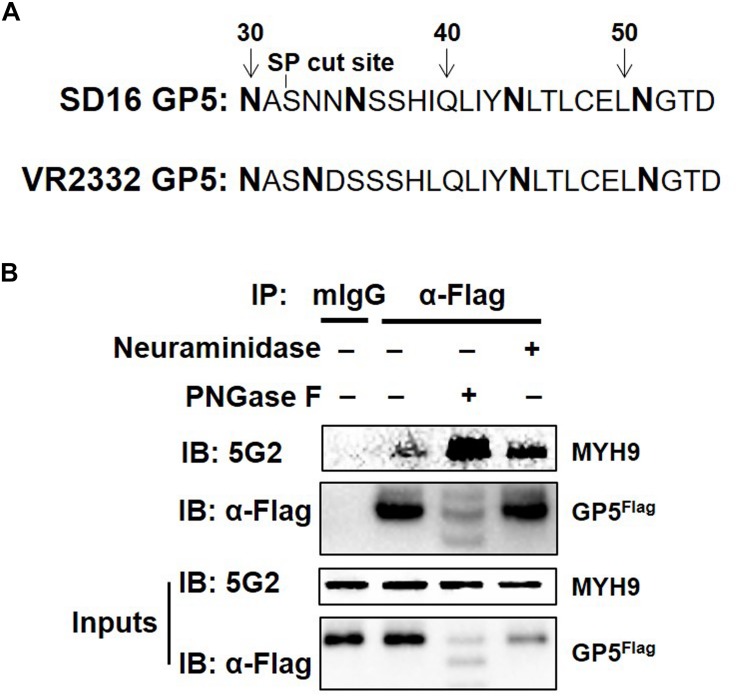
PRRSV GP5-MYH9 interaction is independent of GP5 glycosylation. **(A)** Predicted N-linked glycosylation sites of GP5 from PRRSV SD16 and VR2332 strains. The bold letters are predicted Asparagine linked glycosylation sites. **(B)** Removal of N-glycosylation and sialic acid in SD16 GP5 does not affect the interaction between GP5 with MYH9. Cell lysates of MARC-145-GP5^*Flag*^ cells were either treated with neuraminidase or PNGase F followed by Co-IP using anti-Flag mAb or anti-mouse antibody (mIgG) as an isotype control antibody. The immunoprecipitation proteins were immunoblotted using Mab2-5G2 and anti-Flag mAb.

MYH9 has been identified as the viral receptor or factor for herpes simplex virus-1 (HSV-1) (Arii et al., 2010), severe fever with thrombocytopenia syndrome virus (SFTSV) (Sun et al., 2014), Epstein-Barr virus (EBV), and PRRSV (Xiong et al., 2015; Gao et al., 2016). For PRRSV, GP5 is responsible for the interaction with MYH9 C-terminal region (named PRA) (Gao et al., 2016). PRRSV attachment to permissive cells can be blocked by the treatment of the virus with neuraminidase to modify sialic acid or PNGase F to remove N-linked oligosaccharides from glycoproteins (Delputte and Nauwynck, 2004). As a highly glycosylated protein, N34, N44 and N51 in GP5 are required for the production of infectious PRRSV strain 97-7895 (Ansari et al., 2006). PRRSV-SD16 GP5 has two more putative N-glycosylation sites, at positions N30 and N35 (while at position N33 in VR2332) except at N44 and N51 (Figure 1A). To investigate whether sialic acid or N-glycosylation modification of GP5 affects the form of GP5 and GP5-MYH9 interaction, MARC-145-GP5^*Flag*^ cells were treated with neuraminidase or PNGase F and then lysed to conduct the investigation. Contrary to unaffected in sialic acid modification of GP5 by neuraminidase treatment, PNGase F treatment caused the production of GP5 in different molecular sizes (Inputs in Figure 1B), indicating the de-glycosylation occurring in GP5. Notably, co-immunoprecipitation (Co-IP) results showed that de-glycosylated GP5 did interact with MYH9, indicating that N-glycosylation of GP5 did not participate in the interaction between GP5 and MYH9 (Figure 1B).

### Determination of GP5 Domains Interact With MYH9 C-Terminal Region

**FIGURE 2:**
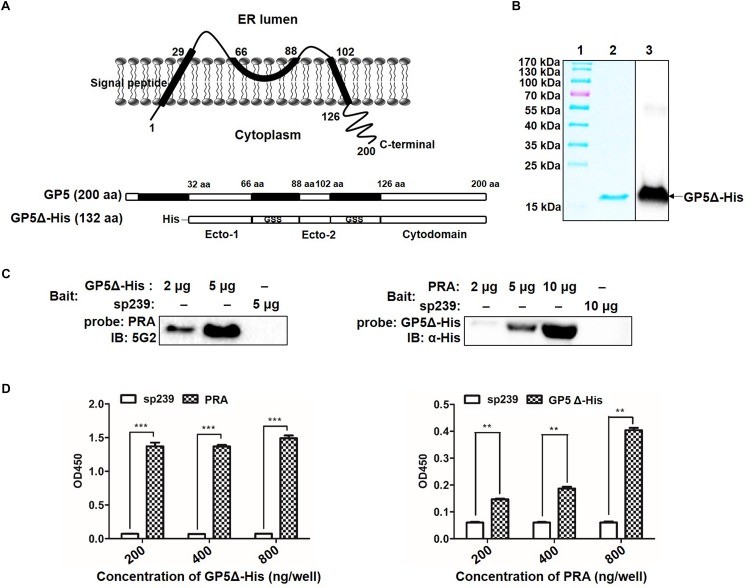
Determination of GP5 domains interact with MYH9 C-terminal region. **(A)** Predicted topological structure PRRSV SD16 GP5 (top panel) and two ectodomains (Ecto-1 and Ecto-2; bottom panel) and a cytoplasmic domain (Cytodomain) were linked with two GSS linkers and labeled with His-tag to form a truncated GP5 protein (designated GP5Δ-His). **(B)** The purified GP5Δ-His protein was confirmed with SDS-PAGE and Western blot analysis. **(C)** Far-Western blot analysis of GP5Δ-PRA interaction. Purified GP5Δ-His (2 μg or 5 μg per lane) and PRA (2 μg, 5 μg, or 10 μg per lane), subjected to SDS-PAGE and transferred to PVDF membrane, were probed with PRA and GP5Δ-His, and then detected with Mab2-5G2 and anti-His mAb, respectively. Recombinant swine hepatitis E virus ORF2 protein (sp239) was used as an irrelevant protein control. **(D)** Indirect ELISA results of GP5Δ-PRA interaction. Purified GP5Δ-His and PRA protein (200, 400, and 800 ng per well) on the solid-phase of the ELISA plate were incubated with PRA and GP5Δ-His, and following detected with Mab2-5G2 and anti-His mAb, respectively. Data are represented as means ± SD. ^∗∗^*P* < 0.01; ^∗∗∗^*P* < 0.001.

Topological analysis of GP5 based on its deduced amino acid sequence from the PRRSV-SD16 GP5 suggested that GP5 contains an N-terminal signal peptide (SP), two ectodomains, two transmembrane domains (TM), and a C-terminal cytoplasmic domain (Figure 2A). Our previous study had demonstrated that PRRSV GP5 expressed in MARC-145 cells interacted with endogenous MYH9 whereby the recombinantly expressed C-terminal domain of MYH9 (PRA) could capture free PRRSV virions *in vitro* (Li et al., 2018), implying a direct interaction between GP5 and PRA. To identify the binding domain of PRRSV GP5 with PRA, a GP5 truncation containing two ectodomains (32-66 aa and 89-102 aa) and a cytoplasmic tail (126-200 aa) (designated GP5Δ-His) was constructed (Figure 2A). We next addressed whether there is a direct interaction between GP5Δ and PRA since MYH9 has been demonstrated to have other interaction partners (Lin et al., 2012). To exclude other adaptor proteins involves in the interaction, recombinant GP5Δ-His protein was produced and purified (Figure 2B) and tested for its direct binding to PRA protein produced as previously described (Li et al., 2018). The results from the far-Western blot and indirect ELISA analyses showed the GP5Δ directly interacted with PRA in a dose-dependent manner with no requirement of other adaptor proteins (Figures 2C,D).

### The GP5 First Ectodomain Interacts With PRA

**FIGURE 3:**
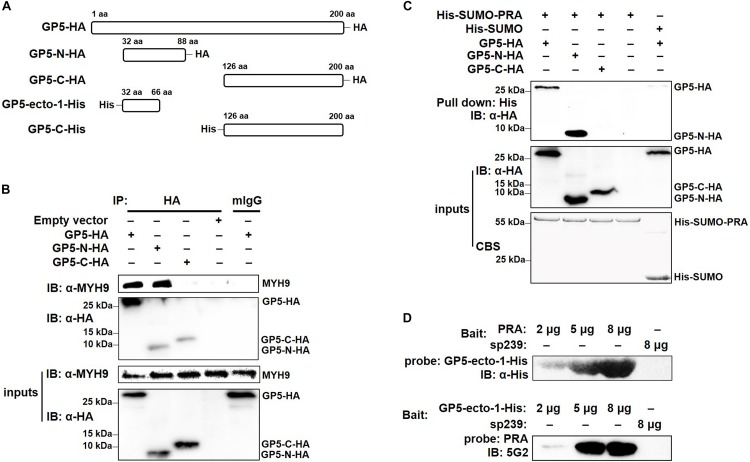
The first ectodomain of GP5 interacts with PRA. **(A)** Schematic diagram of different fragments of GP5. **(B)** Co-IP analysis for interaction between different GP5 domains with endogenous MYH9. Lysates of MARC-145 cells transfected with plasmids encoding GP5-HA, GP5-N-HA, and GP5-C-HA were subjected to Co-IP using anti-HA mAb and the immunoprecipitation were immunoblotted using anti-MYH9 polyclonal Abs and anti-HA mAb. **(C)** His-pull-down analysis for interaction between PRA and different GP5 domain proteins. Purified His-SUMO-PRA or His-SUMO (as a control) proteins were captured with anti-His resin and then incubated with lysates from COS7 cells transfected with plasmids encoding GP5-HA, GP5-N-HA, and GP5-C-HA. The interaction complexes were pull-downed and subjected to the SDS-PAGE and Western blot via immunoblotting using anti-HA mAb. **(D)** Far-western blot analysis of recombinantly expressed GP5-ecto-1 and PRA interaction. Purified PRA and GP5-ecto-1-His (2, 5, or 8 μg per lane) subjected to SDS-PAGE and transferred to PVDF membrane were probed with GP5-ecto-1-His and PRA, and then detected with anti-His mAb and Mab2-5G2, respectively. Protein sp239 was used as an irrelevant protein control.

To determine the GP5-binding domain more precisely, full-length GP5 labeled with an HA tag (GP5-HA), a truncated protein containing the first ectodomain and followed transmembrane domain at the N-terminal end (GP5-N-HA), and a truncated protein containing a cytoplasmic domain at the C-terminal end (GP5-C-HA) were cloned (Figure 3A) and transfected into MARC-145 cells. The results demonstrated that GP5 and GP5-N, but not GP5-C, co-precipitated with endogenous MYH9 (Figure 3B). Since GP5 interacts with PRA, MYH9 C-terminal domain protein (Li et al., 2018), the soluble form of recombinant SUMO-PRA was labeled with a His tag then used for the following studies. First, the interaction between PRA and GP5-N was confirmed that PRA co-precipitated with GP5 and GP5-N, but not with GP5-C, from MYH9-deficient COS7 cells transfected with plasmids enabling expression of GP5, GP5-N, or GP5-C, respectively (Figure 3C). Furthermore, we focused on the first GP5 ectodomain within GP5-N (designated GP5-ecto-1) to investigate its interaction with PRA. Using far-Western blot analysis, purified GP5-ecto-1 recombinant protein was shown to interact with PRA in a dose-dependent specific interaction (Figure 3D).

### Interaction With GP5-Ecto-1 Triggers MYH9 to Form Aggregates

**FIGURE 4:**
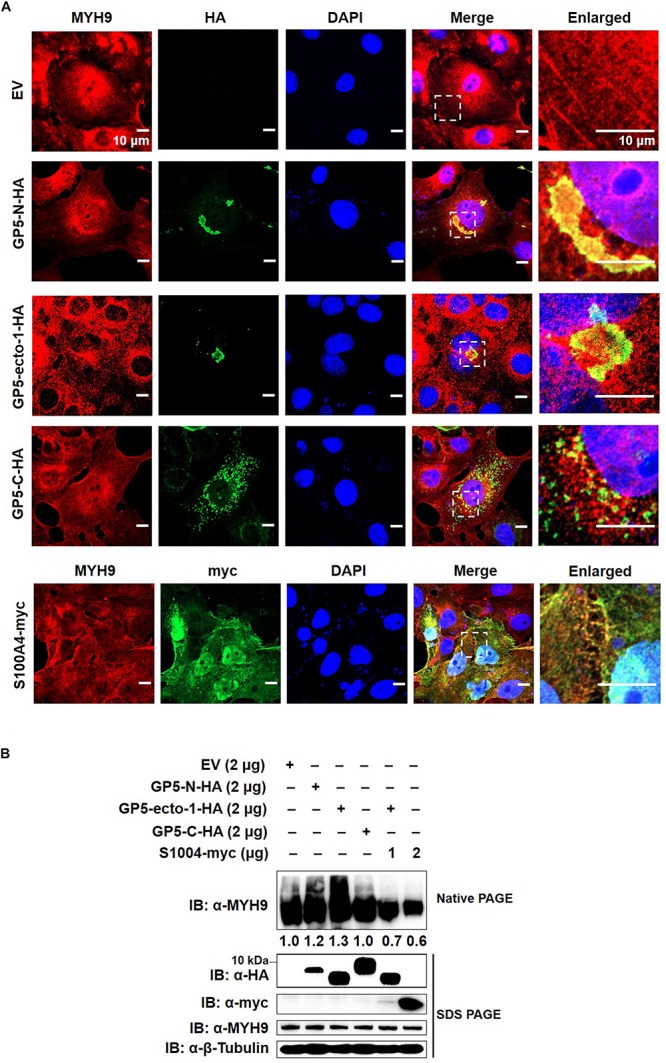
The interaction of GP5-ecto-1 with PRA induces MYH9 aggregation. **(A)** Confocal microscope analysis of co-localization of different GP5 domain proteins with MYH9 and formation of MYH9 aggregates in MARC-145 cells expressing GP5-N-HA, GP5-ecto-1-HA, GP5-C-HA, S100A4-myc, and pCAGEN empty vector (EV). MARC-145 cells were transfected with indicated plasmids for 48 h and followed by fixation. After permeabilization, cells were stained for MYH9 using anti-MYH9 pAb (Red) and for different GP5 fragments or S100A4 using anti-HA mAb or anti-myc mAb (Green). Cellular nuclei were counterstained with DAPI (Blue). Representative images are shown, representing one confocal z-section through the middle of the cells. Bars, 10 μm. **(B)** Native-PAGE for aggregated MYH9 in MARC-145 cells transfected with indicated plasmids as described in panel **(A)** as well as co-transfection of GP5-ecto-1-HA with S100A4-myc. After 48 h transfection, the cells were lysed with NP40 lysis buffer. MYH9 aggregation and total MYH9 expression were analyzed via Native-PAGE and Western blot using anti-MYH9 pAb. Values are normalized to MARC-145 cells transfected with EV and the fold of relative expression was indicated.

To further evaluate the impact of GP5-ecto-1 on the interaction with MYH9, MARC-145 cells were transiently transfected with plasmids encoding HA-labeled GP5-ecto-1, GP5-N, or GP5-C, as well as empty vector (EV) as negative control. The results showed that GP5-N and GP5-ecto-1 co-localized with endogenous MYH9 (Figure 4A) with the Manders’ overlap coefficients of 0.89 ± 0.02 and 0.74 ± 0.02, respectively, compared with 0.56 ± 0.01 for GP5-C. Therefore, expression of GP5-N and GP5-ecto-1 within cells enhanced MYH9 aggregates formation by the direct interaction with MYH9. To further verify whether endogenous MYH9 formed aggregates upon interaction with GP5-ecto-1, cell lysates were subjected to native-PAGE and Western blot analyses. SDS-PAGE revealed that the total amounts of MYH9 in whole cell lysates generated from MARC-145 cells expressing GP5-N, GP5-ecto-1, and GP5-C were comparable. However, under native-PAGE conditions, significantly higher amounts of aggregated MYH9 proteins were observed in cells expressing GP5-N and GP5-ecto-1, as compared with cells expressing EV and GP5-C (Figure 4B), indicating that MYH9 aggregate formation was triggered via MYH9 binding to GP5-ecto-1.

MYH9 plays important roles in cell migration, adhesion, and morphogenesis (Joo and Yamada, 2014; Ma and Adelstein, 2014) through maintenance of the cell polarity by continuous tension and filament assembly of MYH9. We therefore hypothesized that MYH9 aggregation is the basis for filament assembly of MYH9. Since S100 Ca^2+^-binding protein A4 (hereafter referred to as S100A4) can interact with longer myosin fragments to trigger MYH9 filament disassembly (Badyal et al., 2011; Kiss et al., 2012; Ramagopal et al., 2013), S100A4 was cloned and transient transfected into MARC-145 cells to evaluate endogenous MYH9 aggregation. Upon subsequent examination, expressed S100A4 protein was evenly distributed within both nucleus and cytoplasm and that it co-localized with MYH9 within the cytoplasmic compartment (Figure 4A). MYH9 aggregates induced by GP5-PRA interaction were further dissociated by S100A4 produced within cells, as evidenced by a marked decrease in MYH9 aggregation observed using native-PAGE (Figure 4B). This result indicates that formation of MYH9 aggregates resulted from abundant MYH9 filament packing.

### Binding to GP5-Ecto-1 Mediates PRA Filamentous Assembly

**FIGURE 5:**
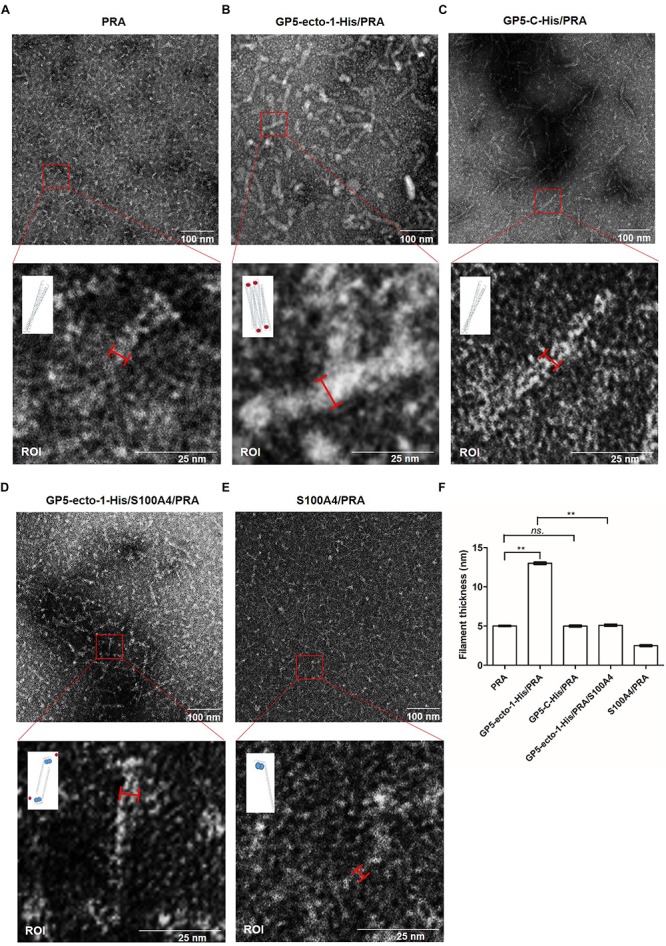
PRA filament assembly *in vitro* is mediated by its interaction with GP5-ecto-1-HA. Negatively stained fields of PRA alone **(A)**, GP5-ecto-1-PRA complex **(B)** and GP5-C-PRA complex **(C)**. Purified PRA (5 mM) alone or pre-incubated with purified GP5-ecto-1-His (10 mM) or GP5-C-His (10 mM) for 1 h at 4°C prior to structure analysis. After centrifuging, each supernatant sample (20 μL) was applied to a carbon-coated grid followed by staining with 1% uranyl acetate. Grids were examined using an H-7650 electron microscope (Hitachi, Tokyo, Japan) operating at 80 kV. Negatively stained fields of GP5-ecto-1-S100A4-PRA complex **(D)** andS100A4-PRA complex **(E)**. Purified S100A4 (10 mM) incubated with complex of GP5-ecto-1-His/PRA or PRA for 1 h at 4°C and sample preparation was performed as described in panel **(A)** Results are representative of three independent experiments. The results of the width of long helical structure were expressed as the mean wide values obtained from ten individual protein complexes ±SD. The schematic diagram of protein complexes was showed in the ROI pictures. PRA filament, GP5-ecto-1-His and S100A4 were represented as long double helix, red circles and blue circles, respectively. Scale bars represent 100 nm in the top panels and 25 nm in the bottom panels. **(F)** The filament width of long helical structures described in **A–E** were assessed as mean ± SD which are representative of ten individual protein complexes. ^∗∗^*P* < 0.01; ns., no significance.

It had been demonstrated previously that the coiled-coil structure located within the C-terminal region of myosin was responsible for formation of bipolar myosin filaments that are required for cellular cargo carriage in cells (Karcher et al., 2002; Zhang et al., 2016). Notably, the PRA region appears to include the last 200 residues of the MYH9 coiled-coil region, which contains the assembly competent domain (ACD) that is crucial for MYH9 filamentous assembly (Elliott et al., 2012). Thus, recombinant PRA alone or PRA after preincubation with purified GP5-ecto-1-His or GP5-C-His recombinant proteins were subjected to structural analysis. Electron microscopy results showed that free PRA protein formed dimeric helical structures with an average width of 5.00 ± 0.15 nm (Figure 5A). Upon binding with GP5-ecto-1, PRA was demonstrated to form filaments of considerable thickness (Figure 5B), as evidenced by a significant increase in average width of long helical structures to 13.00 ± 0.21 nm (*p* < 0.01, Figure 5F). In contrast, interaction with GP5-C did not lead to the emergence of thick PRA filament assemblages (Figures 5C,F), indicating that triggering of assembly of PRA filamentous aggregates only occurs after the addition of GP5-ecto-1.

It had previously been shown that S100A4 protein interacts with the coiled-coil interface of MYH9 and prevents packing of MYH9 filaments (Elliott et al., 2012). Therefore, recombinant S100A4, using methods previously reported (Kiss et al., 2016), was expressed, purified, then incubated with PRA or PRA-GP5-ecto-1 complex to confirm the occurrence of GP5-ecto-1-mediated PRA filament assembly. As shown in Figure 5D, in the presence of S100A4, no thick PRA filament assemblies formed even when PRA was incubated with GP5-ecto-1, with the average width of long helical structures observed to significantly decrease to 5.10 ± 0.15 nm compared to that of PRA-GP5-ecto-1 complex with absence of S100A4 (*p* < 0.01, Figure 5F). In addition, in the presence of S100A4 the average width of helical structures formed free PRA decreased to 2.50 ± 0.15 nm (Figure 5F), indicating that PRA may have dissociated into monomers in the presence of S100A4 (Figure 5E), as demonstrated in a previous report (Elliott et al., 2012).

### Dimerization of PRA Is Required for Its Interaction With PRRSV GP5

**FIGURE 6:**
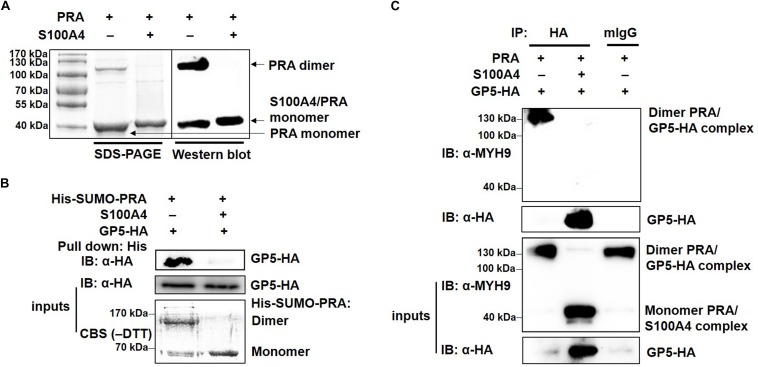
PRRSV GP5 prefers to bind with dimeric PRA to form the interaction. **(A)** SDS-PAGE and Western blot analyses of S100A4-induced disassembly of PRA filaments. PRA (5 mM) alone or pre-incubation with S100A4 (10 mM, contain 3 mM CaCl^2+^) at 4°C for 1 h were immunoblotted using anti-MYH9 pAb in non-reducing condition (without DTT). **(B)** His-pull-down analysis for interaction of GP5 with PRA dimer or monomer. Purified His-SUMO-PRA pretreated with S100A4 or not was bound to anti-His resin and then incubated with lysates from GP5-HA-expressing COS7 cells for pull-down assays. Immunoprecipitated extracts were analyzed by SDS-PAGE and Western blot using anti-HA mAb. CBS: Coomassie blue stain. **(C)** Co-IP analysis for interaction between GP5 and PRA dimer or monomer. Lysates from COS7 cells expressing GP5-HA were incubated with PRA pretreated with or without S100A4 and then subjected to Co-IP using anti-HA mAb and the immunoprecipitation were immunoblotted using anti-MYH9 pAb and anti-HA mAb in non-reducing condition.

MYH9 exists in three different forms: monomer, dimer and polymer (Kiss et al., 2016). To investigate whether PRA polymerization is required for its interaction with PRRSV GP5, recombinant PRA alone or PRA incubated with S100A4 were each subjected to non-reducing SDS-PAGE and Western blot analyses. As shown in Figure 6A, both monomeric and dimeric forms of PRA were detected in the absence of S100A4, whereas only monomeric PRA was observed in the presence of S100A4. Next, the interaction of monomeric and dimeric PRA forms with GP5 were examined using soluble PRA (His-SUMO-PRA) to pull-down GP5-HA stably expressed in COS7 cells. The results showed that only dimeric PRA interacted with GP5, not monomeric PRA released upon addition of S100A4 (Figure 6B). The possibility that the PRA-GP5 interaction was mediated by His or SUMO protein was ruled out by treatment of His-SUMO-PRA with rTEV protease to remove the His-SUMO tag. Furthermore, the direct interaction between GP5 and dimeric PRA was evaluated using co-precipitation under non-reducing conditions, which demonstrated that dimeric PRA-GP5 complexes possessed an approximate MW of 100 kDa, while no interaction between monomeric PRA and GP5 was observed in the presence of S100A4 (Figure 6C). Taken together, these findings suggest that dimerization of PRA is required for its direct interaction with PRRSV GP5.

### MYH9 Aggregation Is Required for PRRSV Internalization

**FIGURE 7:**
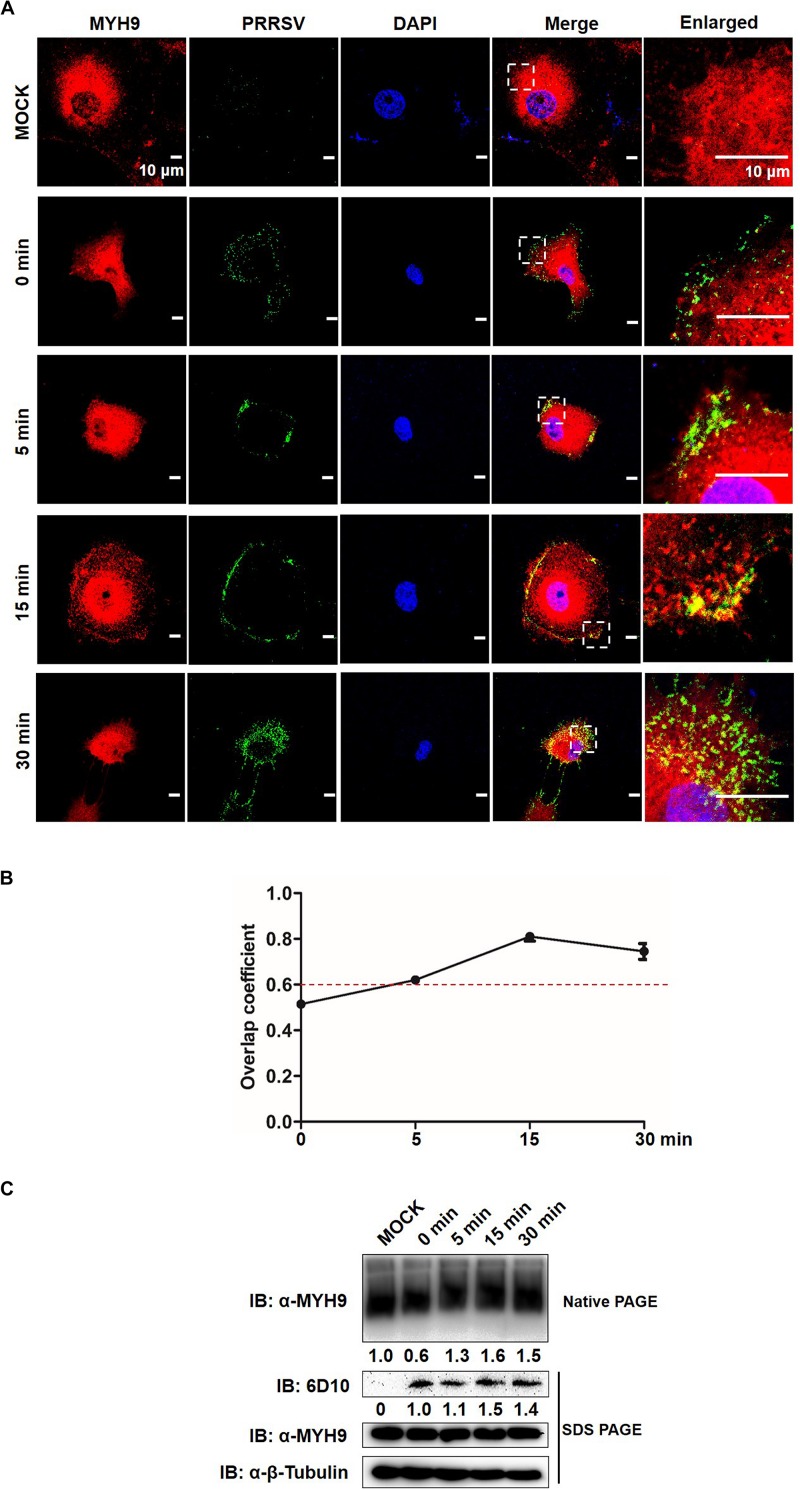
Formed MYH9 aggregates is crucial for virion internalization to MARC-145 cells. **(A)** Co-localization in MARC-145 cells between PRRSV virions and MYH9. After exposure to PRRSV SD16 strain (MOI = 50) at 4°C for 2 h, MARC-145 cells were transferred to 37°C for 0, 5, 15, and 30 min. Cells were fixed, permeabilized and stained with anti-MYH9 pAb (Red) and 6D10 (mAb against PRRSV N protein, in house, Green) to visualize distribution of endogenous MYH9 and viral particles. Representative images are shown, representing one confocal z-section through the middle of the cells. Bars, 10 μm. **(B)** The co-localization of MYH9 and PRRSV described in **(A)** was assessed by determination of Manders’ overlap coefficient using Image-Pro Plus software. The Mean of Manders’ overlapcoefficient ±SD are representative of three individual enlarged pictures. **(C)** Native-PAGE for aggregated MYH9 and Western blot for internalized PRRSV virions in MARC-145 cells during PRRSV internalization. MARC-145 cells were incubated with SD16 at MOI = 50 at 4°C for 2 h following by shifting to 37°C, and then harvested at indicated time points (0, 5, 15, 30 min). Cells were lysed and subjected to native-PAGE and Western blot using anti-MYH9 pAb and anti-PRRSV N mAb (6D10). Values are normalized to uninfected MARC-145 cells and the fold of relative expression was indicated.

Since the data described above indicate that a direct interaction between GP5 and PRA led to PRA filamentous assembly, we then asked whether endogenous MYH9 forms filamentous assemblies during its interaction with PRRSV GP5; if yes, what is the function of MYH9 aggregation in the context of PRRSV infection. To this question, MARC-145 cells were inoculated with PRRSV SD16 strain (MOI = 50), incubated at 4°C for 2 h, then transferred to 37°C to trigger virus endocytosis followed by assessment of MYH9 filament assembly at indicated time points. Co-localization of PRRSV virions with endogenous MYH9 and MYH9 filament assembly into aggregates were both observed by 15 min after the temperature shift to 37°C (Figure 7A) with an overlap coefficient of >0.6 (Figure 7B). Next, quantification of MYH9 aggregates was evaluated using native-PAGE and Western blot analysis. Indeed, after temperature shift to 37°C, markedly increased MYH9 aggregation was detected at 5 min by 1.3-fold and continued to increase to 1.6-fold and 1.5-fold at 15 and 30 min, respectively. Notably, after the temperature shift, increased PRRSV N protein levels, to 1.5-fold and 1.4-fold initial levels, were detected at 15 min and 30 min, respectively (Figure 7C).

**FIGURE 8:**
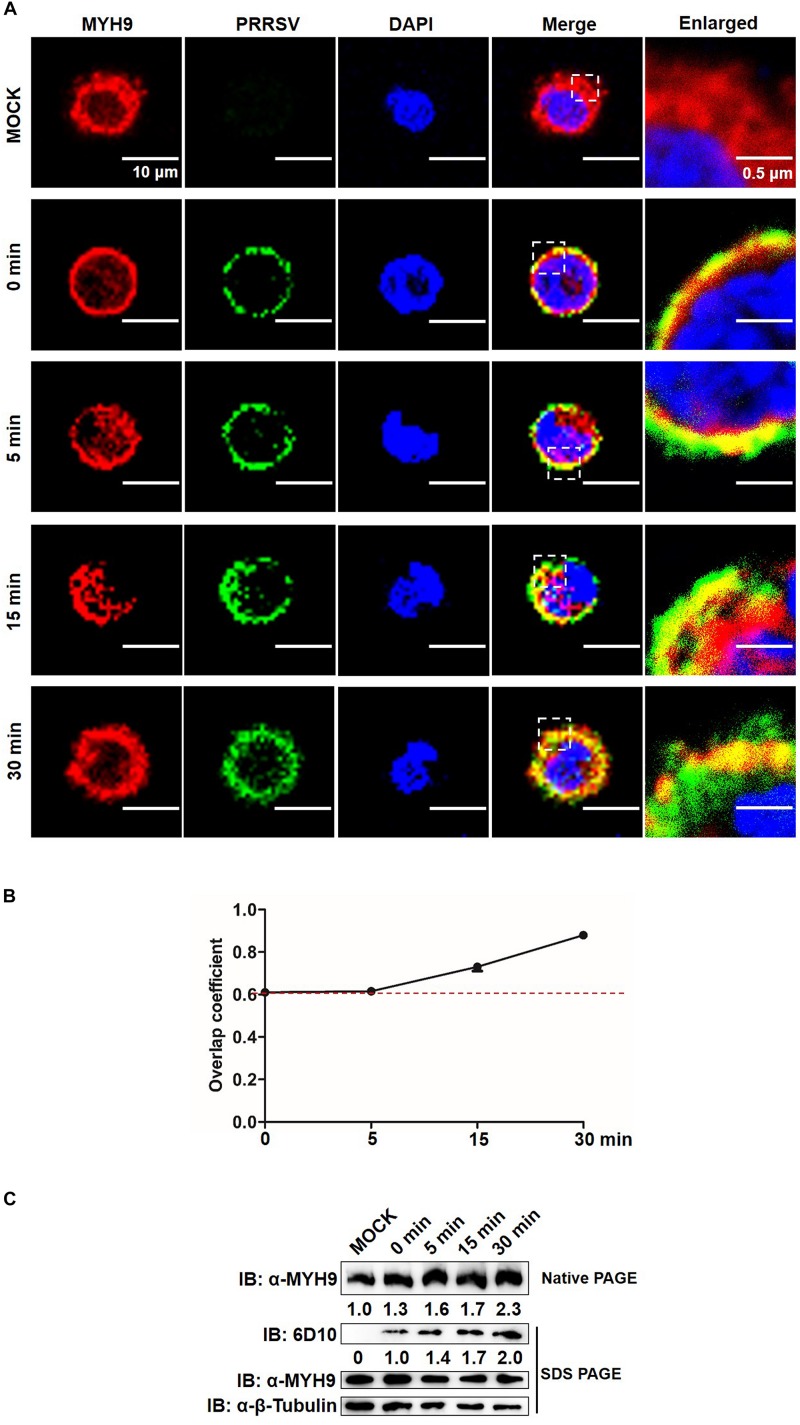
MYH9 aggregation facilitates PRRSV internalization into PAMs. **(A)** Co-localization in PAMs between PRRSV virions and MYH9. PAMs were incubated with PRRSV SD16 strain (MOI = 50) at 4°C for 2 h following by shifting to 37°C, and then fixed, permeabilized and subjected to immunofluorescence staining of MYH9 (Red) and PRRSV virions (Green). Representative images are shown, representing one confocal z-section through the middle of the cells. Bars, 10 μm.**(B)** The co-localization of MYH9 and PRRSV described in panel **(A)** was assessed by determination of Manders’ overlap coefficient using Image-Pro Plus software. The Mean of Manders’ overlap coefficient ± SD are representative of three individual enlarged pictures. **(C)** Native-PAGE for aggregated MYH9 and Western blot for internalized PRRSV virions in PAMs during PRRSV internalization. After exposure to SD16 at MOI = 50 at 4°C for 2 h, PAMs were transferred to 37°C for 0, 5, 15, and 30 min. Whole cell lysates of PAMs were harvested and subjected to native-PAGE and Western blot using anti-MYH9 pAb and anti-PRRSV N mAb (6D10). Values are normalized to uninfected PAMs and the fold of relative expression was indicated.

We further tested whether MYH9 filamentous packing, through its interaction with PRRSV GP5, induced a similar pattern of PRRSV internalization by PAMs. Similar to results for MARC-145 cells, endogenous MYH9 co-localized with PRRSV virions and formed MYH9 aggregates 15 min after a temperature shift to 37°C (Figure 8A) with an overlap coefficient >0.6 (Figure 8B). Furthermore, increased amounts of both MYH9 aggregates and internalized PRRSV virions were also detected in infected PAMs with increases of 2.3-fold and 2.0-fold, respectively, by 30 min after a temperature shift (Figure 8C). Along with the results from Figures 5, 6, it showed a positive correlation between MYH9 aggregate formation and PRRSV internalization. Together, these findings indicated that MYH9 aggregation is required for PRRSV internalization.

### Disassembly of MYH9 Aggregates Regulated by S100A4 Significantly Diminishes PRRSV Internalization

**FIGURE 9:**
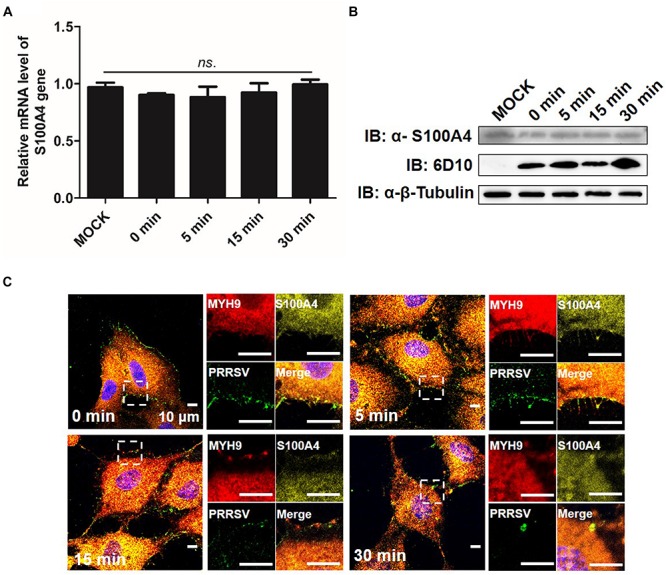
Expression of endogenous S100A4 protein is unchanged on RNA and protein levels, and mainly distributes in cytoplasm during PRRSV internalization. **(A)** S100A4 mRNA level was analyzed by qPCR. After exposure to PRRSV SD16 strain (MOI = 50) at 4°C for 2 h, MARC-145 cells were transferred to 37°C for 0, 5, 15, and 30 min. Whole cell lysates were harvested and mRNA levels were calculated relative to known amounts of template and normalized to GAPDH expression. Data are represented as means ± SD. ns, no significant. **(B)** S100A4 protein levels were analyzed by Western blot analysis. MARC-145 cells were incubated with SD16 at MOI = 50 at 4°C for 2 h following by shifting to 37°C, and then cells were lysed and subjected to Western blot using anti-S100A4 pAb and anti-PRRSV-N Mab-6D10. **(C)** Confocal microscope analysis of distribution of MYH9, S100A4 and PRRSV virions in cells. MARC-145 cells were incubated with SD16 (MOI = 50) at 4°C for 2 h following by shifting to 37°C, and then fixed, permeabilized and stained with anti-MYH9 pAb (Red), anti-S100A4 pAb (Yellow) and 6D10 (Green). Images are representative one of three independent experiments.

It was previously shown that both mRNA and protein level indicators of S100A4 expression were upregulated in migrating cells (Kim and Helfman, 2003). Here, we investigated the expression and distribution pattern of endogenous S100A4 in MARC-145 cells during PRRSV internalization. From 0 to 30 min after a temperature shift to 37°C, no significant changes in mRNA and protein expression levels of S100A4 were observed (Figures 9A,B). MYH9 aggregates on the cell membrane were clearly observed at 15 min (Figure 9C), a result consistent with data depicted in Figure 7A. Meanwhile, endogenous S100A4 protein was mainly found within the cytoplasm and did not co-localize with MYH9 aggregates during PRRSV internalization (Figure 9C). Therefore, these results, when considered together, may indirectly explain why MYH9 aggregate formation triggered by interaction with PRRSV GP5 within the permissive cells facilitates PRRSV internalization.

**FIGURE 10:**
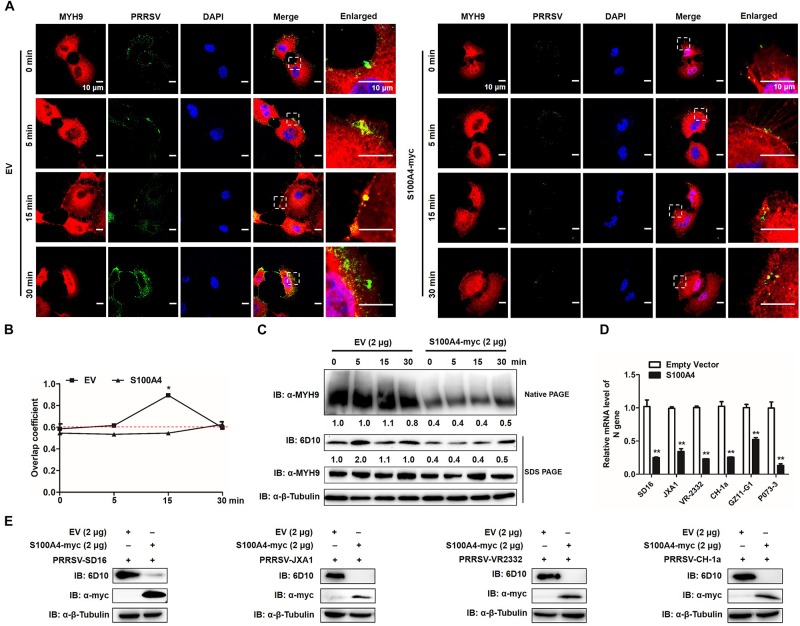
GP5-ecto-1-PRA interaction-induced PRA aggregation correlates PRRSV internalization and sequential viral replication. **(A)** Co-localization in MARC-145 cells between PRRSV and MYH9. After exposure to PRRSV (MOI = 50) at 4°C for 2 h, MARC-145 cells expressing S100A4-myc or transfected with pCAGEN empty vector (EV) were transferred to 37°C for 0, 5, 15, and 30 min. Cells were fixed, permeabilized and subjected to immunofluorescence staining of MYH9 (Red) and PRRSV virions (Green). Images are representative one of three independent experiments. Bars, 10 μm. **(B)** Assessment of co-localization of MYH9 and PRRSV virions. The Mean of Manders’ overlap coefficient ± SD are representative of three individual enlarged pictures. ^∗^*P* < 0.05. **(C)** Cell treatment was performed as described in panel **(A)**, followed by cell lysed and subjected to native-PAGE and Western blot using anti-MYH9 pAb and anti-PRRSV N mAb (6D10). Values are normalized to PRRSV-infected cells with EV-transfection that harvested at 0 min after temperature shift, and the fold of relative expression was indicated. **(D)** MARC-145 cells expressing S100A4-myc or transfected with pCAGEN empty vector (EV) were inoculated with indicated PRRSV strains at 50 MOI for 15 min. The RNA level of PRRSV-N expression was detected by qPCR. Values are normalized to EV-transfected cells with PRRSV infection. ^∗∗^*P* < 0.01. **(E)** Cell treatment was performed as described in panel **(D)** and then cells were washed with PBS buffer and cultured for 24 h. Virus replication was measured via Western blot using anti-PRRSV N mAb (6D10).

To further verify whether formation of MYH9 filamentous aggregates facilitated PRRSV internalization or alternatively resulted from the viral internalization process, MARC-145 cells were transfected with plasmid enabling recombinant S100A4 expression or with empty vector (EV). Transfected cells were then infected by addition of PRRSV and incubated with virus at 4°C for 2 h. Next, transfected cells were incubated at 37°C then assessed for MYH9 aggregate formation and PRRSV internalization at indicated time points. No co-localization of PRRSV virions and endogenous MYH9 was observed and fewer MYH9 aggregates were observed in the presence of S100A4 (Figure 10A). Moreover, the Mander’s overlap coefficient for EV-transfected cells was significantly higher than coefficient values for S100A4-expressing cells during PRRSV internalization (15 min after the temperature shift) (0.89 ± 0.007 vs. 0.545 ± 0.007, *P* = 0.018, Figure 10B), demonstrating disruption of co-localization of PRRSV virions with endogenous MYH9 after expression of S100A4 within MARC-145 cells during PRRSV internalization.

To further quantify MYH9 aggregates and internalized PRRSV virions, each was evaluated in S100A4- or EV-transfected MARC-145 cells at indicated time points after a temperature shift to 37°C. Markedly decreased MYH9 aggregation within S100A4-expressing cells versus control was detected at 0 min as a 0.4-fold decrease that remained at the same level for 30 min after the temperature shift. Meanwhile, a similar reduction of PRRSV N protein was observed in MARC-145 cells transfected with S100A4 at each indicated time point after the triggering of endocytosis (Figure 10C), indicating virus internalization by permissive cells was markedly diminished due to decreased MYH9 aggregation mediated by artificially induced S100A4 expression.

Similar inhibition of viral internalization was further confirmed in S100A4-transfected MARC-145 cells inoculated with heterogeneous PRRSV isolates, including HP-PRRSV isolates (SD16 and JXA1), North American classical PRRSV (VR2332), Chinese isolated strain (CH-1a) and two *PRRSV-1* Chinese isolates GZ11-G1 and P073-3, as determined by qPCR-based detection of internalized PRRSV virions (Figure 10D). Consequently, infection by heterogeneous PRRSV strains was markedly inhibited in S100A4-transfected MARC-145 cells compared to inhibition observed for EV-transfected cells measured by Anti-N Mab-6D10 except for *PRRSV-1* strains due to the antigenic variation of N protein (Figure 10E). Collectively, these findings support the conclusion that MYH9 filament aggregation is required for PRRSV internalization and subsequent viral infection.

## Discussion

MYH9, also known as non-muscle myosin heavy chain 9, is originally identified as a motor protein involved in cell migration, adhesion, and morphogenesis (Joo and Yamada, 2014). The amino-terminal head domain of MYH9 possesses ATPase activity and contains binding sites for both actin and myosin light chains (Sellers, 2000). However, the roles played by MYH9 during viral infection or infections involving other pathogens have only been recently investigated. To date, it has been reported that herpes simplex virus-1 (HSV-1), thrombocytopenia syndrome virus (SFTSV), Epstein-Barr virus (EBV), and PRRSV utilize MYH9 as a necessary factor for infection of permissive cells (Arii et al., 2010; Sun et al., 2014; Xiong et al., 2015). Notably, at least one viral protein present on virion surfaces of each of the abovementioned viruses has been reported to interact with MYH9, including gB of HSV-1, Gn of SFTSV, and gH/gL of EBV (Arii et al., 2010; Sun et al., 2014; Xiong et al., 2015).

Regarding the case of PRRSV, it has been proposed that GP2, GP3, and GP4 proteins located within PRRSV virions are able to form multiprotein complexes with the CD163 cellular interaction partner as an essential step for viral infectivity and receptor binding (Lee et al., 2004; Wissink et al., 2005; Das et al., 2011). However, numerous studies have also implicated PRRSV GP5 in virus infectivity, including our previous reports identifying PRRSV GP5 protein as the potential interaction partner for MYH9 in PRRSV-permissive cells (Gao et al., 2016; Li et al., 2018). In fact, this result aligns with investigations by other groups that demonstrated that the GP5-MYH9 interaction participates in intercellular spread of PRRSV to neighboring cells via nanotubes (Guo et al., 2016). Yet other studies have shown that interruption of the GP5-MYH9 interaction by recombinantly expressed PRA proteins could block PRRSV infection *in vitro*, suggesting the PRA domain located within the C-terminal portion of MYH9 is responsible for binding to PRRSV GP5 (Li et al., 2018). Similar observations for HSV-1 blocking of MYH9 have been reported, with specific antibody resulting in inhibition of HSV-1 infection of susceptible cells (Arii et al., 2010). Therefore, it appears that the interaction between PRRSV GP5 and MYH9 is essential for completion of the PRRSV replication cycle.

Full-length MYH9 is an actin-binding protein that mainly exists within the cytoplasm (Guo et al., 2016). The role of MYH9 as a potential receptor or co-receptor for viral infection, as well as mechanisms of interaction between viral proteins and MYH9, are poorly understood. Available data gained from studies of HSV, EBV, and PRRSV suggest that redistribution of MYH9 from cytoplasm to plasma membrane appears to be a common step required for virion internalization when endocytosis of virion-bound cells is triggered by a temperature switch to 37°C (Arii et al., 2010; Sun et al., 2014; Xiong et al., 2015; Gao et al., 2016). Specifically, PRRSV virions are internalized by permissive cells via a clathrin-mediated endocytosis step (Nauwynck et al., 1999) that requires participation of unconventional myosin proteins (such as MYO6) for uncoating of endocytic vesicles (Aschenbrenner et al., 2004). We therefore speculate that the GP5-MYH9 interaction may participate in internalization of PRRSV virions via endocytosis, with cytoplasmic uncoating of endocytic vesicles containing PRRSV virions necessary for initiation of viral entry. This speculation is consistent with observations that transient expression of porcine CD163 alone did not confer PRRSV susceptibility to COS7 cells, which lack endogenous MYH9 expression, unless MYH9 expression is artificially induced (Gao et al., 2016).

To locate the GP5 domain that participates in the GP5-MYH9 interaction, truncated GP5 proteins were generated based on the predicted amino acid sequence model for GP5. The model predicts the presence of a signal peptide, ectodomains, transmembrane domains, cytoplasmic domain, and secondary structures. Based on our results, it appears that the first GP5 ectodomain (GP5-ecto-1) is involved in the MYH9 interaction and induces MYH9 aggregation in MARC-145 cells, with glycosylation or sialic acid modification of GP5 playing no role in the interaction. Using this *in vitro* model, electron microscopic analysis demonstrated that the interaction between GP5-ecto-1 and PRA (within the MYH9 C-terminal domain) induced PRA filamentous aggregate formation. These results are consistent with the proposed function of the last 200 residues of the coiled-coil region containing the MYH9 ACD that is responsible for regulating myosin filament assembly (Elliott et al., 2012). Moreover, GP5-ecto-1-induced aggregation of PRA is consistent with the polymerization and aggregation behavior of MYH9, as observed in virus-bound MARC-145 cells after triggering of endocytosis-mediated virion internalization by a temperature switch to 37°C.

Mammalian MYH9 includes activated monomers and polymers that perform physiological roles *in vivo*, such as initiation of contractile system assembly (Shutova et al., 2014). In this study, PRRSV-induced polymerization and aggregation of MYH9 were simultaneously confirmed by confocal microscopy and native-PAGE combined with Western blot analysis. It appears that polymerization-based MYH9 aggregation is required for PRRSV internalization and may be induced by PRRSV GP5 protein via the GP5-MYH9 interaction. By contrast, S100A4, a calcium binding regulatory protein that is a member of the S100 protein family, exhibits a favorable high-affinity binding interaction with MYH9 that induces disassembly of MYH9 filaments (Du et al., 2012; Elliott et al., 2012). Therefore, S100A4 may effectively inhibit MYH9 aggregation, as supported by its efficacy in inhibiting infection of permissive cells by all PRRSV isolates tested. Most importantly, it appears that GP5 preferably interacts with PRA dimers, which contain the ACD of MYH9. Since mRNA and protein levels of S100A4 were previously demonstrated to be upregulated during cell recruitment and migration (Dulyaninova et al., 2018), we measured expression levels and cellular distribution of endogenous S100A4. It showed unchanged mRNA and protein expression levels were observed with no co-localization of S100A4 with MYH9 aggregates during PRRSV internalization, implying that S100A4 does not participate in MYH9-mediated endocytosis. However, S100A4 might transfer ‘call-for-help’ signals to healthy cells during the middle and late stages of PRRSV infection, as evidenced by markedly increased abundance of S100A4 in PRRSV-infected cells from 12 to 48 hpi (Guo et al., 2018).

Recently, our latest report also demonstrated that MYH9 interact with CD163 N-terminal domain which contributes to PRRSV internalization (Hou et al., 2019). Combined together, our result underlined a model linking the GP2/GP3/GP4-CD163 (initiating virus-receptors binding) and GP5/MYH9 (triggering endocytosis) together. It appears that attachment of PRRSV virions to cell surfaces was initiated via interactions between CD163 and GP2a/GP4 complexes. Then MYH9 is transported to the cell surface, where it becomes accessible to binding by PRRSV GP5 protein and also interacting with CD163 N-terminal domain to enhance virion internalization. Next, the interaction between PRRSV GP5 and MYH9 (preferably dimerized MYH9) induces polymerization and aggregation of MYH9 to allow myosin filament assembly and acquisition of motor activity (Sellers and Umemoto, 1989; Shutova et al., 2014). Motor activity then acts as an essential step in endocytosis-mediated PRRSV virion internalization that occurs prior to initiation of PRRSV replication in susceptible cells (Wang et al., 2014). Besides, MYH9 also involved in recognition of sialic acids on PRRSV to suppress proinflammatory responses via the DAP12-Syk pathway (Liu et al., 2019), further promote replication of PRRSV.

## Conclusion

In conclusion, our results indicate that PRRSV entry is highly regulated by virus structural protein GP5 and cellular MYH9. In this work, a direct interaction between GP5 and MYH9 was confirmed that induces polymerization/aggregation of MYH9, a necessary step for myosin filament assembly that facilitates entry of PRRSV virions into permissive cells. Meanwhile, S100A4 blocking of MYH9 aggregation depends on myosin filament disassembly and exhibits broad inhibition of a variety of isolates of *PRRSV-1* and *PRRSV-2*. Our results not only provide novel insights for understanding the molecular basis of PRRSV virion internalization by susceptible cells, but also suggest that this mechanism of virus internalization could be employed as a potential antiviral target for use in prevention of PRRSV infection.

## Data Availability Statement

All datasets generated for this study are included in the manuscript.

## Author Contributions

BX and GH performed the research, analyzed the data, and drafted the manuscript. GZ, JH, and LL contributed to the construction of cell lines. LW, LZ, XH, and XR contributed to the production of recombinant proteins. YN, YM, and QZ revised the manuscript. CW, JW, and E-MZ conceived the study, carried out additional analyses and finalized the manuscript. All authors contributed to the revising of the manuscript.

## Conflict of Interest

The authors declare that the research was conducted in the absence of any commercial or financial relationships that could be construed as a potential conflict of interest.
